# Design, Synthesis and Biological Evaluation of 6,7-Disubstituted-4-phenoxyquinoline Derivatives Bearing Pyridazinone Moiety as c-Met Inhibitors

**DOI:** 10.3390/molecules23071543

**Published:** 2018-06-26

**Authors:** Xiaobo Liu, Jianlan Kou, Zhen Xiao, Fajuan Tian, Jiayi Hu, Pengwu Zheng, Wufu Zhu

**Affiliations:** 1 Jiangxi Provincial Key Laboratory of Drug Design and Evaluation, School of Pharmacy, Jiangxi Science & Technology Normal University, Nanchang 330013, China; 18790658261@163.com (X.L.); xz950420@163.com (Z.X.); 15679170508@163.com (F.T.); 15797865216@163.com (J.H.); 2 Teaching and Research Department of Mathematics and Chemistry, Nanchang Health School, Nanchang 330006, China; hjy15717910426@163.com

**Keywords:** quinoline derivatives, pyridazinone, c-Met, antiproliferative activity

## Abstract

Deregulation of the receptor tyrosine kinase mesenchymal epithelial transition factor (MET) has been implicated in several human cancers and is an attractive target for small molecule drug discovery. Herein, a series of 6,7-disubstituted-4-phenoxyquinoline derivatives bearing pyridazinone derivatives were designed, synthesized and evaluated for their enzymatic inhibitory activity against c-Met kinase and cellular potency against A549, HepG2, and MCF-7 cell lines. Eight of them are equal to more active than positive control Foretinib against one or more cell lines and enzyme. The most promising compound **53** showed superior activity to Foretinib, which possessed excellent c-Met kinase inhibition on a singledigital nanomolar level (IC_50_ = 0.6 nM), and cancer cells of A549 (IC_50_ = 0.003 µM), HepG2 (IC_50_ = 0.49 µM) and MCF-7 cells (IC_50_ = 0.006 µM). The result of AO single staining indicated that compound **53** could induce remarkable apoptosis of HepG2 cell.

## 1. Introduction

The c-mesenchymal-epithelia transition factor (c-Met), known as hepatocyte growth factor receptor (HGFR), belongs to the receptor tyrosine kinases (RTKs) subfamily [1,2]. The MET receptor tyrosine kinase is activated by binding to its ligand HGF, resulting in the dimerization of the receptor. Then, Tyr1234/1235 is autophosphorylated, and activates the kinase domain and downstream multiple signaling pathways (including RAS/MAPK and PI3K/AKT), which promotes cell proliferation, survival, movement, invasion, angiogenesis and morphogenesis [3,4]. Aberrant c-Met signaling activation due to gene amplification, rearrangement, point mutations, and autocrine or paracrine HGF stimulation has been implicated in many types of human malignancies [5,6,7,8,9]. In addition, HGF/c-Met over-activation is involved in mediating intrinsic or acquired resistance to target therapies. For example, amplification of the Met has been detected in 20% of acquired resistance to epidermal growth factor receptor (EGFR) inhibitors [10,11]. Therefore, the c-Met axis has emerged as an attractive target for targeted cancer therapy.

**Figure 1:**
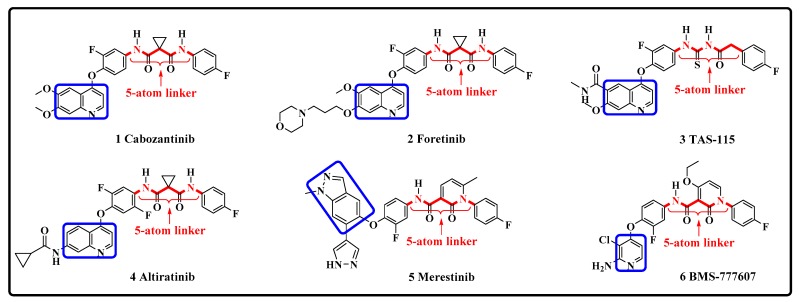
The representative small-molecule c-Met kinase inhibitors.

In recent years, significant progress has been made in the development of small molecule c-Met kinase inhibitors. Cabozantinib (**1** XL-184) is the first multitarget small molecule c-Met kinase inhibitor approved by the FDA for the treatment of medullary thyroid carcinoma on 29 November 2012 [12]. Foretinib (**2** GSK1363089), the first small molecule oral inhibitor to enter clinical trials, is in clinical stage II currently [13]. Many other kinds of c-Met inhibitor are also reported, such as TAS-115, Altiratinib, Merestinib, BMS-777607 (**3**–**6**, Figure 1) [14,15,16,17,18].

Because of its excellent efficacy in vivo, favorable pharmacokinetics and preclinical safety profiles, Foretinib was chosen as an extension of our work on the development of novel potent c-Met inhibitors. And many researches have shown that almost all Foretinib derivatives containing 5-atom linker between the aminophenoxy at C-4 position of quinoline and aryl group, which we called “5-atom regulation” exhibit excellent activity. The ‘5-atom regulation’ have two obvious structural characteristics. One is that there is six chemical bonds distance between aminophenoxy at C-4 position of quinoline moiety and aryl group moiety, and the other one is that the “six chemical bonds linker” contains hydrogen, oxygen, and nitrogen atoms which could form hydrogen-bond donor or acceptor (Figure 1) [19].

**Figure 2:**
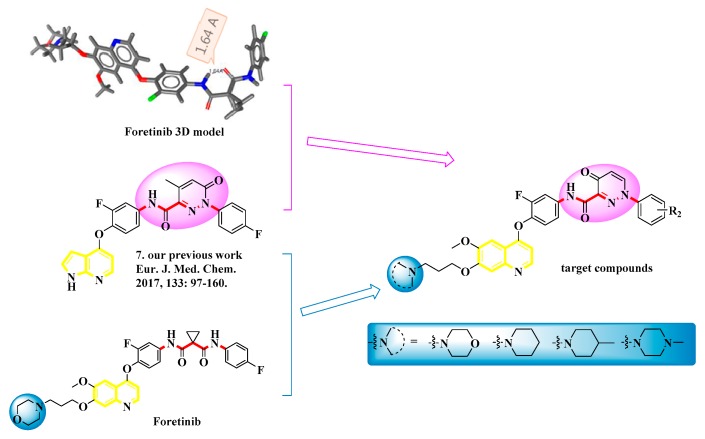
Structures and design strategy for target compounds.

In our previous research, most of these compounds showed potent activity, especially the most promising compound **7** (Figure 2) with the enzyme activity IC_50_ values in the nanomole level [20]. The SARs and docking study exhibited that 6-oxo-pyridazinone moiety may be benefit to the in vitro activity. From the 3D model of Foretinib, we can know that Foretinib contain an intermolecular hydrogen (distance = 1.64 A°) in the 5-atom linker. And inspired by compound **7**, 4-oxo-pyridazinone was introduced into 5-atom linker moiety to limit the conformation deeply.

The biological activity of compound **7** incorporating pyrrolo[2,3-b]pyridine was not optimal. Therefore, reference was made to the target compound Foretinib, which retains the 3-carbon tether at the 7 position of quinolone. In order to further increase the solubility of the compound to increase activity, the morpholinyl group was replaced by other water-soluble substituents, including piperidinyl, 4-methylpiperidinyl and 4-methylpiperazine, to observe the effects of the different cyclic tertiary amino groups on activity of the novel compounds. Furthermore, various substituents (R_2_) were introduced at the phenyl ring to investigate their effects on activity. Accordingly, we designed a novel series of 6,7-disubstituted-4-phenoxy quinoline derivatives bearing pyridazinone moiety.

The target compounds were evaluated for their enzymatic inhibitory activity against c-Met kinase and cellular potency in human liver cancer (HepG2), human breast cancer (MCF-7) and human lung adenocarcinoma (A549) cell lines [21]. Moreover, AO single staining and docking studies were presented in this paper as well.

## 2. Results and Discussion

**Scheme 1:**
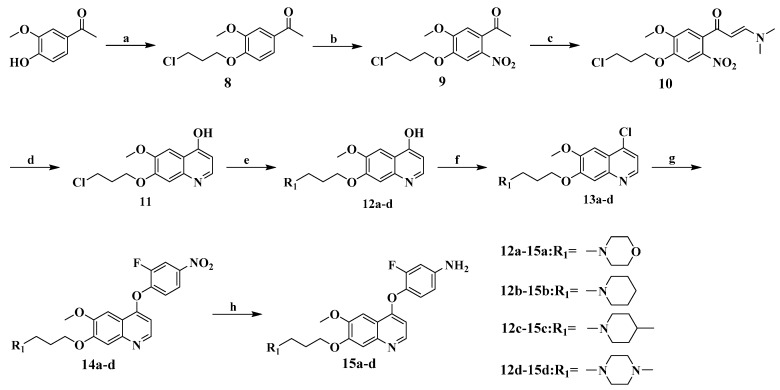
Reagents and conditions: (**a**) Br(CH_2_)_3_Cl, acetone, 0 °C, 30 min, rt, 12 h; (**b**) 98% HNO_3_, CH_2_Cl_2_, 0 °C, 4 h; (**c**) DMF-DMA, toluene, 110 °C, 10 h; (**d**) Fe powder, AcOH, rt, 30 min, 80 °C, 2 h; (**e**) secondary amines, CH_3_CN, 85 °C, 10 h; (f) POCl_3_, 85 °C, 6 h; (**g**) 2-fluoro-4-nitrophenol, PhCl, 140 °C, 30 h; (**h**) Fe powder, NH_4_Cl(cat.), EtOH/H_2_O, reflux, 5 h.

The synthesis of the key intermediates of 6,7-disubstituted-4-phenoxyquinolines **15a**–**d** was illustrated in Scheme 1. The commercially available starting material 1-(4-hydroxy-3-methoxyphenyl) ethanone was alkylated with 1-bromo-3-chloropropane in acetone under basic condition to provide compound **8**, which was converted to nitro compound **9** using fuming nitric acid as nitration reagent in dichloromethane at −20 °C for 6 h. Next, condensation of **9** with dimethyl formamide dimethyl acetal (DMF-DMA) in refluxing toluene afforded yellow solid **10**, which was reduced and cyclized using glacial acetic acid and iron powder to provide hydroxy-quinoline **11** with a high yield and purity in a single step. Substitution of **11** with excessive secondary amines (piperidine, 4-methyl piperidine, morpholine, and pyrrolidine) in acetonitrile at reflux provided intermediates **12a**–**d**, which were treated with phosphorus oxychloride to afford chloro-quinoline **13a**–**d**, respectively. Further, the obtained compounds **13a**–**d** were etherified with 2-fluoro-4-nitrophenol in chlorobenzene to obtain purified compounds **14a**–**d**, which were reduced using iron powder and catalytic amounts of ammonium chloride in ethanol to obtain amides **15a**–**d [22]**.

**Scheme 2:**
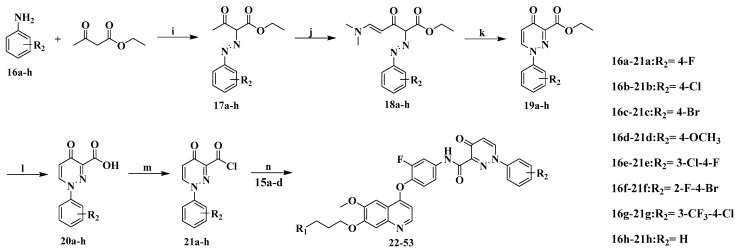
Reagents and conditions: (**i**) sodium nitrite, EtOH, ethyl acetoactate, 0 °C, 30 min; (**j**) DMF-DMA, 100 °C, reflux, 6–10 h; (**k**) EtOH, 10% NaOH, 80 °C, 1 h; (**l**) sodium carbonate, EtOH/H_2_O (5:1), 80 °C, 3-5 h; (**m**) oxalyl chloride, DMF, CH_2_Cl_2_, rt, 5 min; (**n**) DIPEA, CH_2_Cl_2_, rt, 0.5 h.

The target compounds **22**–**53** were prepared as illustrated in Scheme 2 Substituted anilines **16a**–**h** were diazotized and then reacted with ethyl aceto-acetate to get compounds **17a**–**h**, which condensed with DMF-DMA to yield compounds **18a**–**h**. Following by cyclization reaction, hydrolysis reaction and chlorination reaction, **21a**–**h** were obtained. Finally, reaction of amides **15a**–**d** with acyl chlorides **21a**–**h** promoted by DIPEA in dichloromethane at room temperature yielded the target compounds **22**–**53**.

### 2.1. Biological Evaluation

Taking Foretinib as reference compound, the target compounds (**22**–**53**) were evaluated for the activity against c-Met kinase and the cytotoxicity against three cancer cell lines A549, HepG2, MCF-7 by 3-(4,5-dimethylthiazolyl-2)-2, 5-diphenyltetrazolium bromide (MTT) cell proliferation assay. The results expressed as IC_50_ values were summarized in Table 1, and the values were the average of at least two independent experiments.

As illustrated in Table 1, most target compounds showed moderate to significant cytotoxic activities against one or more tested cancer cells with potencies in the single digit micromole range, which suggested that the introduction “5-atom linker” to 4-oxo-pyridazinone framework maintained the potent cytotoxic activity. Six compounds (**37**, **41**, **42**, **43**, **52** and **53**) exhibited promising cytotoxicity with IC_50_ values ranging from 0.002 to 1.03 µM, which was comparable to that of Foretinib. It is worth noting that the most significant candidate compound **53** showed significant activity with IC_50_ values of 0.003 µM, 0.49 µM, 0.006 µM for A549, HepG2 and MCF-7 cells, respectively, which were higher than those of Foretinib against A549 cell (0.26 µM), HepG2 cell (0.84 µM), MCF-7 cell (2.76 µM).

Target compounds were further determined for c-Met kinase activity through homogenous time-resolved fluorescence (HTRF) assays. As shown in Table 1, most target compounds exhibited moderate to excellent c-Met enzymatic potency with IC_50_ values ranging, suggesting that the inhibition of c-Met may be a main mechanism for the antitumor activity of the prepared compounds. Four compounds **41** (0.9 nM), **42** (1.2 nM), **52** (0.8 nM) and **53** (0.6 nM) exhibited promising enzymatic activity, which were more active than reference compound Foretinib (1.8 nM).

The structure-activity relationship (SAR) was commenced by the morpholinyl group was replaced by other water-soluble substituents, including piperidinyl, 4-methylpiperidinyl and 4-methylpiperazine. The pharmacological activity data indicated that the R_1_ group affected the activity dramatically. The compounds (**22**–**29**) substituted with morpholine have slight reduced the cytotoxicity activity against A549, HepG-2 and MCF-7 cell lines than the compounds (**30**–**53**) substituted with other three water-soluble groups (piperidine, 4-methylpiperidine and 4-methylpiperazine). Furthermore, the cell lines data revealed a clear preference for activity when the R_1_ group was 4-methyl piperazinyl group, which indicated that a more water-soluble cyclic tertiary amino group at the C-7 position of quinoline contributed to the potency of the target compounds. Such as, the IC_50_ values of compound **52** were (0.8 nM, 0.04 µM) against c-Met kinases and A549 cell lines, which were clearly lower than that of compound **28** (382.0 nM, 5.25 µM), compound **36** (11.0 nM, 0.79 µM) and compound **44** (19.0 nM, 0.15 µM).

Further investigations were performed to study the effect of different substituents on the phenyl ring on the cytotoxic activity. In general, it seems to be that target compounds with no less than one electron-withdrawing groups (EWGs) showed better in vitro activity, and the aryl group substituted with double-EWGs is more preferred. For instance, the enzymatic potency of double-EWGs compounds **34** (2.0 nM), **35** (8.0 nM) and **36** (11.0 nM), were clearly lower than that of mono-EWGs compounds **30** (17.0 nM), **31** (78.0 nM) and **32** (246.0 nM). These data also showed that when there is only one EWGs (4-F, 4-Cl, 4-Br), the compounds substituted with the substituent of increasing electrophilic ability were beneficial to the antiproliferative activity. In addition, compounds without substituent group on aryl group exhibit the best activity, such as compounds **29**, **37** and **53**. However, compound **45** is an exception, compounds substituted with the substituent of increasing electrophilic ability were beneficial to the antiproliferative activity.

### 2.2. Acridine Orange (AO) Staining Is Used to Analyze Cell Morphological Changes of the HepG2 Cells

**Figure 3:**
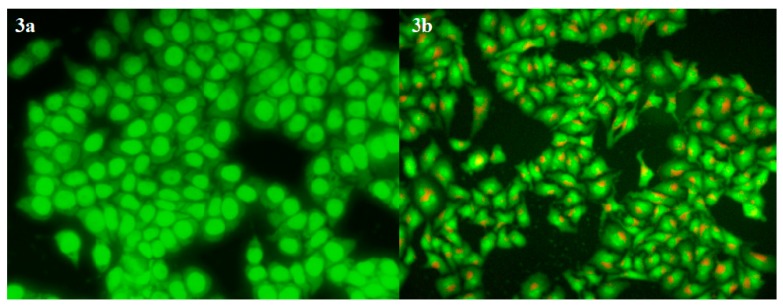
The cell morphology of HepG2 cells of the control and test groups. (**a**) HepG2 cells treated without any compounds; (**b**) HepG2 cells were treated with 0.01 µM for 12 h; Changes of nuclear morphology were determined by AO staining.

Potential compound **53** was selected for validation and evaluation of inhibition of cell proliferation and induction of apoptosis in HepG2 cells. AO staining fluorescence analysis results showed that compared to the control group (Figure 3a), compound **53** (Figure 3b) significantly inhibited HepG2 cells proliferation and induced apoptosis at the concentration of 0.01 μM. Moreover, from the results of the inverted fluorescence microscopy, it can be observed that the cells in the control group (Figure 3a) were full and the edge was clear, which was added without nothing. And HepG2 cells were treated with compound **53**, it can be seen in (Figure 3b) that the HepG2 cells showed obviously shrink into chromatin, with sharp edges and consistent with the phenomenon of apoptosis. 

### 2.3. Molecular Docking Study

In order to explore the binding mode of the target compounds with the c-Met active site, molecular docking simulation studies were performed using AutoDock vina v1.02 (The Scripps Research Institute, La Jolla, CA, USA) and Discovery Studio 3.5 (Dassault Systèmes BIOVIA, San Diego, CA, USA). Based on the in vitro inhibition results, we selected compound **53**, our best c-Met inhibitor in this study, as the ligand example, and the structure of c-Met was selected as the docking model (PDB ID code: 3LQ8).

**Figure 4:**
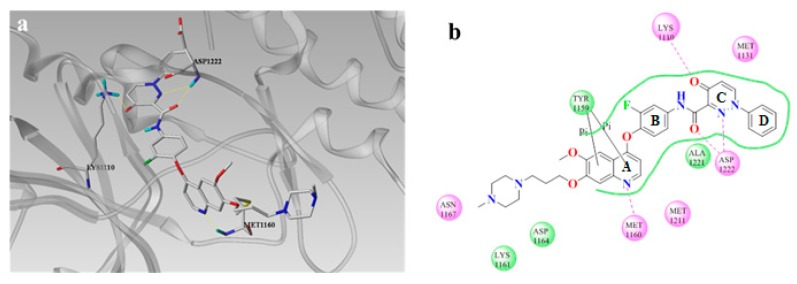
The docking mode of **53** with c-Met. (**a**) The 3D interaction map between the **53** and c-Met, the hydrogen bonds was colored with yellow; (**b**) 2D depiction of the ligand-protein interaction.

The binding modes of compound **53** and c-Met were depicted in Figure 4a,b. In the binding mode, compound **53** is potently bound to the active binding site of c-Met via four hydrogen bonds and one pi–pi interactions. The the quinoline group (A ring) formed one hydrogen bond and one pi–pi interaction with MET 1160 and TYR 1159 respectively. Then the oxygen atom of the 5-atom linker moiety and the nitrogen atom of the pyridazinone moiety (C ring) formed bidentate hydrogen bond with the amino hydrogen of ASP 1222. In addition, the oxygen atom of the pyridazinone moiety (C ring) formed one hydrogen bond with the amino hydrogen of LYS 1110. In general, these results of the molecular docking study showed that 6,7-disubstituted-4-phenoxyquinoline derivatives bearing pyridazinone moiety could act synergistically to interact with the active binding site of c-Met, suggested that compound **53** may be a potential inhibitor of c-Met.

## 3. Experimental Section

### 3.1. General Information

All melting points were obtained on a Büchi Melting Point B-540 apparatus (Büchi Labortechnik, Flawil, Switzerland) and were uncorrected. Nuclear Magnetic Resonance (NMR) spectra were performed using Bruker 400 MHz spectrometers (Bruker Bioscience, Billerica, MA, USA) with tetramethylsilane (TMS) as an internal standard. Mass spectra (MS) were taken in electrospray ionization (ESI) mode on Agilent 1100 Liquid chromatography–mass spectrometry (LC-MS) (Agilent, Palo Alto, CA, USA). Thin layer chromatography (TLC) analysis was carried out on silica gel plates GF254 (Qingdao Haiyang Chemical, Qingdao, China). All materials were obtained from commercial suppliers and used without purification, unless otherwise specified. Yields were optimized. 

### 3.2. Chemistry

#### 3.2.1. Preparation of 3-Fluoro-4-(6,7-disubstituted quinolin-4-yloxy)anilines **15a**–**d**

The preparation of the key intermediates **15a**–**d** has been illustrated in detail in our previous work [22], and so the synthesis method would not be listed here.

#### 3.2.2. Preparation of Compounds **17a**–**h**

Appropriate substituted anilines **16a**–**h** (0.101 mol) was added to the mixture solution of hydrogen chloride/water (1:1, 10 mL), sodium nitrite (0.1 mol) and stirred for 5 min at 0 °C, respectively. Then the mixture was added to the ethanol solution which ammonium acetate (1 mol) and ethyl acetoacetate (0.1 mol) were dissolved in. The reaction was monitored by TLC until completed. Finally the solution was filtered and washed with a plenty of water to give a yellow solid.

#### 3.2.3. Preparation of Compounds **18a**–**h**

The yellow solid **17a**–**h** (0.115 mol) was dissolve in the DMF-DMA (50 mL), heated from 0 °C to 100 °C and stirred for 3–4 h. After the reaction was completed, poured the mixture to the petroleum ether and filtered to obtain the compounds **18a**–**h**.

#### 3.2.4. Preparation of Compounds **19a**–**h**

The compounds **18a**–**h** were dissolved in ethanol at 80 °C. Then 10% NaOH was added in the reaction solution slowly, refluxed for 1 h and monitored by TLC. The solution was concentrated in vacuum, the residue was resolved with dichloromethane (300 mL), washed with brine (60 mL), dried over anhydrous Na_2_SO_4_, and concentrated in vacuum to give the yellow solids **19a**–**h**. 

#### 3.2.5. Preparation of Compounds **20a**–**h**

Ethanol (100 mL) and water (10 mL) were added to a flask containing compounds **19a**–**h** (0.01 mol), and then sodium hydroxide (0.02 mol) was added portionwise, and the reaction was carried out at room temperature. As the reaction progresses, a large amount of precipitate is generated. Water was added to dissolve the precipitate, and then activated carbon was added to remove the pigment and remove it from the aqueous solution. The filtrate was extracted and the pH adjusted to 3-4 with hydrochloric acid to give a white solid **20a**–**h**.

#### 3.2.6. Preparation of Compounds **21a**–**h**

The compounds **20a**–**h** (1 mmol) and appropriate DMF (0.1 mmol) were dissolved in dichloromethane, then appropriate oxalyl chloride was added slowly and monitored by TLC. The solution was used for next step without further purification.

#### 3.2.7. Preparation of Compounds **22**–**53**

A solution of phenylpyrdazinone carbonyl chloride **21a**–**h** (0.82 mmol) in dichloromethane (10 mL) was added drop-wise to a solution of aniline **15a**–**d** (0.41 mmol) and diisopropylethylamine (0.49 mmol) in dichloromethane (10 mL) in an ice bath. Upon completion of the addition, the reaction mixture was removed from the ice bath and placed in room temperature for 30 min and monitored by TLC. The mixture was concentrated in vacuum to yield **22**–**53** which were recrystallized by isopropanol.

*N-(3-Fluoro-4-((6-methoxy-7-(3-morpholinopropoxy)quinolin-4-yl)xy)phenyl)-1-4-fluorophenyl)-4-oxo-1,4-dihydropyridazine-3-carboxamide* (**22**). Light yellow solid. 37.2% yield, m.p: 152.3–153.5 °C. ^1^H NMR (400 MHz, DMSO) δ 12.02 (s, 1H), 8.99 (d, *J* = 7.8 Hz, 1H), 8.48 (d, *J* = 5.2 Hz, 1H), 8.02 (d, *J* = 12.7 Hz, 1H), 7.86 (dd, *J* = 8.9, 4.6 Hz, 2H), 7.55 (d, *J* = 12.2 Hz, 2H), 7.49 (dt, *J* = 15.2, 7.6 Hz, 3H), 7.40 (s, 1H), 6.93 (d, *J* = 7.8 Hz, 1H), 6.49 (d, *J* = 5.2 Hz, 1H), 4.20 (t, *J* = 6.4 Hz, 2H), 3.95 (s, 3H), 3.59 (t, *J* = 4.2 Hz, 4H), 2.47 (d, *J* = 7.0 Hz, 2H), 2.40 (s, 4H), 2.02–1.94 (m, 2H). ^13^C NMR (101 MHz, DMSO) δ 169.09, 162.83, 159.92, 159.17, 154.74, 152.30, 151.93, 149.58, 148.81, 147.50, 146.37, 142.03, 139.41, 137.05, 136.95, 124.33, 123.87, 123.79, 120.46, 116.62, 116.38, 114.45, 108.53, 102.09, 101.50, 99.02, 66.66, 66.18(2C), 55.76, 54.79, 53.34(2C), 25.66. TOF MS ES+ (*m*/*z*): (M + H)^+^, calcd for C_34_H_31_F_2_N_5_O_6_: 644.2321, found, 644.2304.

*1-(4-Chlorophenyl)-N-(3-fluoro-4-((6-methoxy-7-(3-morpholinopropoxy)quinolin-4-yl)oxy)phenyl)-4-oxo-1,4-dihydropyridazine-3-carboxamide* (**23**). Light yellow solid. 36.2% yield, m.p: 138.2–139.1 °C. ^1^H NMR (400 MHz, DMSO) δ 11.95 (s, 1H), 9.02 (d, *J* = 7.9 Hz, 1H), 8.49 (d, *J* = 5.0 Hz, 1H), 8.02 (d, *J* = 12.8 Hz, 1H), 7.86 (d, *J* = 8.5 Hz, 2H), 7.70 (d, *J* = 8.5 Hz, 2H), 7.55 (d, *J* = 10.6 Hz, 2H), 7.50 (t, *J* = 8.7 Hz, 1H), 7.44 (s, 1H), 6.93 (d, *J* = 7.8 Hz, 1H), 6.51 (d, *J* = 5.0 Hz, 1H), 4.26 (s, 2H), 3.96 (s, 3H), 3.78 (s, 4H), 3.04 (s, 4H), 2.50–2.48 (m, 2H), 2.20 (s, 2H). TOF MS ES+ (*m*/*z*): (M + H)^+^, calcd for C_34_H_31_ClFN_5_O_6_: 660.2025, found, 660.2009.

*1-(4-Bromophenyl)-N-(3-fluoro-4-((6-methoxy-7-(3-morpholinopropoxy)quinolin-4-yl)oxy)phenyl)-4-oxo-1,4-dihydropyridazine-3-carboxamide* (**24**). Light yellow solid. 37.9% yield, m.p: 125.7–127.1 °C. ^1^H NMR (400 MHz, DMSO) δ 12.03 (s, 1H), 9.12 (d, *J* = 7.8 Hz, 1H), 8.58 (d, *J* = 5.2 Hz, 1H), 8.11 (d, *J* = 12.8 Hz, 1H), 7.91 (q, *J* = 8.6 Hz, 4H), 7.66 (d, *J* = 9.9 Hz, 2H), 7.60 (t, *J* = 8.7 Hz, 2H), 7.52 (s, 1H), 7.03 (d, *J* = 7.8 Hz, 1H), 6.60 (d, *J* = 5.1 Hz, 1H), 4.31 (d, *J* = 5.9 Hz, 2H), 4.05 (s, 3H), 3.76 (s, 4H), 2.76 (s, 4H), 2.60 (s, 2H), 2.15 (s, 2H). TOF MS ES+ (*m*/*z*): (M + H)^+^, calcd for C_34_H_31_BrFN_5_O_6_: 704.1520, found, 704.1501.

*N-(3-Fluoro-4-((6-methoxy-7-(3-morpholinopropoxy)quinolin-4-yl)oxy)phenyl)-1-(4-methoxyphenyl)-4-oxo-1,4-dihydropyridazine-3-carboxamide* (**25**). White solid. 38.1% yield, m.p: 153.8–155.2 °C. ^1^H NMR (400 MHz, DMSO) δ 12.18 (s, 1H), 8.95 (d, *J* = 7.8 Hz, 1H), 8.69 (d, *J* = 5.8 Hz, 1H), 8.08 (d, *J* = 12.6 Hz, 1H), 7.77–7.67 (m, 3H), 7.59 (dd, *J* = 20.1, 9.7 Hz, 3H), 7.15 (d, *J* = 9.0 Hz, 2H), 6.92 (d, *J* = 7.8 Hz, 1H), 6.81 (d, *J* = 4.7 Hz, 1H), 4.33 (s, 2H), 4.02 (s, 3H), 3.84 (s, 3H), 3.49 (s, 4H), 3.12 (s, 4H), 2.50–2.49 (m, 2H), 2.35 (s, 2H). ^13^C NMR (101 MHz, DMSO) δ 169.48, 160.52, 159.82, 159.74, 155.18, 152.73, 152.11, 149.98, 149.21, 147.60, 146.46, 142.42, 137.63, 136.81, 124.77, 123.44(2C), 121.10, 117.15, 115.14(2C), 109.25, 109.04, 102.70, 101.95, 99.60, 66.58, 63.96, 56.30, 56.10(2C), 54.20, 51.79(2C), 23.59. TOF MS ES+ (*m*/*z*): (M + H)^+^, calcd for C_35_H_34_FN_5_O_7_: 656.2521, found, 656.2503.

*1-(3-Chloro-4-fluorophenyl)-N-(3-fluoro-4-((6-methoxy-7-(3-morpholinopropoxy)quinolin-4-yl)oxy)phenyl)-4-oxo-1,4-dihydropyridazine-3-carboxamide* (**26**). Pure white solid. 35.1% yield, m.p: 138.1–140.6 °C. ^1^H NMR (400 MHz, DMSO) δ 11.84 (s, 1H), 8.93 (d, *J* = 7.3 Hz, 1H), 8.39 (d, *J* = 4.6 Hz, 1H), 8.05 (s, 1H), 7.92 (d, *J* = 12.9 Hz, 1H), 7.77 (s, 1H), 7.61 (t, *J* = 8.8 Hz, 1H), 7.47 (d, *J* = 11.6 Hz, 2H), 7.44–7.38 (m, 1H), 7.31 (s, 1H), 6.85 (d, *J* = 7.5 Hz, 1H), 6.40 (d, *J* = 4.8 Hz, 1H), 4.11 (s, 2H), 3.87 (s, 3H), 3.50 (s, 4H), 2.37 (d, *J* = 6.5 Hz, 2H), 2.31 (s, 4H), 1.90 (d, *J* = 5.5 Hz, 2H). ^13^C NMR (101 MHz, DMSO) δ 169.67, 160.19, 159.65, 158.56, 156.09, 155.23, 152.78, 152.45, 150.11, 149.26, 147.94, 146.91, 142.28, 140.13, 137.45, 124.72, 124.21, 122.72, 120.84, 118.39, 118.17, 117.17, 115.01, 109.09, 102.62, 99.57, 67.19, 66.70(2C), 56.26, 55.29, 53.85(2C), 26.19. TOF MS ES+ (*m*/*z*): (M + H)^+^, calcd for C_34_H_30_ClF_2_N_5_O_6_: 678.1931, found, 678.1908.

*1-(4-Bromo-2-fluorophenyl)-N-(3-fluoro-4-((6-methoxy-7-(3-morpholinopropoxy)quinolin-4-yl)oxy)phenyl)-4-oxo-1,4-dihydropyridazine-3-carboxamide* (**27**). White solid. 35.7% yield, m.p: 113.7–115.1 °C. ^1^H NMR (400 MHz, DMSO) δ 11.82 (s, 1H), 8.75 (d, *J* = 7.7 Hz, 1H), 8.47 (d, *J* = 5.2 Hz, 1H), 7.97 (d, *J* = 10.3 Hz, 2H), 7.76 (t, *J* = 8.3 Hz, 1H), 7.69 (d, *J* = 8.7 Hz, 1H), 7.53 (s, 2H), 7.51–7.45 (m, 1H), 7.40 (s, 1H), 6.90 (d, *J* = 7.8 Hz, 1H), 6.48 (d, *J* = 5.0 Hz, 1H), 4.19 (t, *J* = 6.1 Hz, 2H), 3.95 (s, 3H), 3.59 (d, *J* = 3.7 Hz, 4H), 2.46 (t, *J* = 6.9 Hz, 2H), 2.39 (s, 4H), 2.04–1.93 (m, 2H). TOF MS ES+ (*m*/*z*): (M + H)^+^, calcd for C_34_H_30_BrF_2_N_5_O_6_: 722.1426, found, 722.1403.

*1-(4-Chloro-3-(trifluoromethyl)phenyl)-N-(3-fluoro-4-((6-methoxy-7-(3-morpholinopropoxy)quinolin-4-yl)oxy)phenyl)-4-oxo-1,4-dihydropyridazine-3-carboxamide* (**28**). Light yellow solid. 47.3% yield, m.p: 203.5–204.7 °C. ^1^H NMR (400 MHz, DMSO) δ 11.75 (s, 1H), 9.02 (d, *J* = 7.8 Hz, 1H), 8.37 (d, *J* = 5.3 Hz, 1H), 8.19 (s, 1H), 8.06 (d, *J* = 7.1 Hz, 1H), 7.91 (d, *J* = 9.6 Hz, 2H), 7.49–7.42 (m, 2H), 7.39 (d, *J* = 9.3 Hz, 1H), 7.30 (s, 1H), 6.85 (d, *J* = 7.9 Hz, 1H), 6.39 (d, *J* = 5.2 Hz, 1H), 4.10 (s, 2H), 3.85 (s, 3H), 3.49 (s, 4H), 2.38–2.37 (m, 2H), 2.30 (s, 4H), 1.88 (s, 2H). ^13^C NMR (101 MHz, DMSO) δ 169.17, 159.80, 159.18, 154.74, 152.28, 151.90, 149.60, 148.83, 148.05, 146.35, 141.80, 141.62, 136.87, 136.41, 133.06, 130.37, 126.69, 124.31, 120.91, 120.86, 120.19, 116.70, 114.52, 108.83, 108.60, 102.17, 99.07, 66.61, 65.84(2C), 55.80, 54.67, 53.09(2C), 25.32. TOF MS ES+ (*m*/*z*): (M + H)^+^, calcd for C_35_H_30_ClF_4_N_5_O_6_: 728.1899, found, 728.1875.

*N-(3-Fluoro-4-((6-methoxy-7-(3-morpholinopropoxy)quinolin-4-yl)oxy)phenyl)-4-oxo-1-phenyl-1,4-dihydropyridazine-3-carboxamide* (**29**). Pure white solid. 39.7% yield, m.p: 118.7–119.8 °C. ^1^H NMR (400 MHz, DMSO) δ 12.00 (s, 1H), 9.03 (d, *J* = 7.8 Hz, 1H), 8.47 (d, *J* = 5.2 Hz, 1H), 8.02 (d, *J* = 12.7 Hz, 1H), 7.82 (d, *J* = 7.7 Hz, 2H), 7.63 (t, *J* = 7.2 Hz, 2H), 7.59–7.47 (m, 4H), 7.40 (s, 1H), 6.93 (d, *J* = 7.9 Hz, 1H), 6.49 (d, *J* = 5.3 Hz, 1H), 4.20 (t, *J* = 6.1 Hz, 2H), 3.95 (s, 3H), 3.59 (s, 4H), 2.50–2.48 (m, 2H), 2.40 (s, 4H), 1.99 (d, *J* = 6.6 Hz, 2H). TOF MS ES+ (*m*/*z*): (M + H)^+^, calcd for C_34_H_32_FN_5_O_6_: 626.2415, found, 626.2396.

*N-(3-Fluoro-4-((6-methoxy-7-(3-(piperidin-1-yl)propoxy)quinolin-4-yl)oxy)phenyl)-1-(4-fluorophenyl)-4-oxo-1,4-dihydropyridazine-3-carboxamide* (**30**). Light yellow solid. 43.4% yield, m.p: 149.4–151.1 °C. ^1^H NMR (400 MHz, DMSO) δ 12.03 (s, 1H), 9.00 (d, *J* = 7.8 Hz, 1H), 8.48 (d, *J* = 5.1 Hz, 1H), 8.03 (d, *J* = 12.5 Hz, 1H), 7.87 (dd, *J* = 8.6, 4.3 Hz, 2H), 7.56 (d, *J* = 10.6 Hz, 2H), 7.50 (dd, *J* = 16.0, 7.8 Hz, 3H), 7.41 (s, 1H), 6.94 (d, *J* = 7.8 Hz, 1H), 6.50 (d, *J* = 5.0 Hz, 1H), 4.20 (d, *J* = 6.1 Hz, 2H), 3.96 (s, 3H), 2.51 (s, 2H), 2.42 (s, 4H), 2.00 (d, *J* = 6.7 Hz, 2H), 1.53 (s, 4H), 1.41 (s, 2H). ^13^C NMR (101 MHz, DMSO) δ 169.09, 162.82, 160.37, 159.92, 159.17, 154.74, 152.29, 151.93, 149.59, 148.80, 147.49, 146.38, 142.02, 139.40, 124.31, 123.87, 123.78, 120.46, 116.62(2C), 116.38, 114.46, 108.77, 108.53, 102.09, 99.02, 66.77, 55.76, 54.98, 53.97(2C), 25.91, 25.39(2C), 23.94. TOF MS ES+ (*m*/*z*): (M + H)^+^, calcd for C_35_H_33_F_2_N_5_O_5_: 642.2528, found, 642.2511.

*1-(4-Chlorophenyl)-N-(3-fluoro-4-((6-methoxy-7-(3-(piperidin-1-yl)propoxy)quinolin-4-yl)oxy)phenyl)-4-oxo-1,4-dihydropyridazine-3-carboxamide* (**31**). White solid. 42.7% yield, m.p: 158.2–159.6 °C. ^1^H NMR (400 MHz, DMSO) δ 12.05 (s, 1H), 9.11 (d, *J* = 7.8 Hz, 1H), 8.57 (d, *J* = 5.1 Hz, 1H), 8.11 (d, *J* = 12.4 Hz, 1H), 7.95 (d, *J* = 8.6 Hz, 2H), 7.80 (d, *J* = 8.6 Hz, 2H), 7.70–7.55 (m, 3H), 7.49 (s, 1H), 7.03 (d, *J* = 7.8 Hz, 1H), 6.58 (d, *J* = 4.9 Hz, 1H), 4.27 (d, *J* = 6.0 Hz, 2H), 4.05 (s, 3H), 2.52 (t, *J* = 6.8 Hz, 2H), 2.44 (s, 4H), 2.06 (d, *J* = 6.0 Hz, 2H), 1.60 (s, 4H), 1.48 (s, 2H). ^13^C NMR (101 MHz, DMSO) δ 169.65, 160.56, 159.75, 155.31, 152.57, 150.20, 149.38, 148.49, 146.95, 142.19, 142.17, 142.16, 137.63, 133.58, 130.20(2C), 124.87, 123.61(2C), 120.95, 117.25, 115.04, 109.35, 109.12, 102.69, 99.63, 67.44, 56.36, 55.65, 54.67(2C), 26.68, 26.17(2C), 24.70. TOF MS ES+ (*m*/*z*): (M + H)^+^, calcd for C_35_H_33_ClFN_5_O_5_: 658.2233, found, 658.2209.

*1-(4-Bromophenyl)-N-(3-fluoro-4-((6-methoxy-7-(3-(piperidin-1-yl)propoxy)quinolin-4-yl)oxy)phenyl)-4-oxo-1,4-dihydropyridazine-3-carboxamide* (**32**). Light yellow solid. 48.3% yield, m.p: 141.3–143.4 °C. ^1^H NMR (400 MHz, DMSO) δ 11.94 (s, 1H), 9.02 (d, *J* = 7.8 Hz, 1H), 8.49 (d, *J* = 5.2 Hz, 1H), 8.02 (d, *J* = 12.7 Hz, 1H), 7.84 (d, *J* = 9.0 Hz, 2H), 7.79 (d, *J* = 8.5 Hz, 2H), 7.56 (s, 2H), 7.50 (t, *J* = 8.8 Hz, 1H), 7.43 (s, 1H), 6.93 (d, *J* = 7.9 Hz, 1H), 6.51 (d, *J* = 5.0 Hz, 1H), 4.23 (s, 2H), 3.96 (s, 3H), 2.80 (s, 4H), 2.12 (s, 2H), 1.64 (s, 4H), 1.47 (s, 2H), 1.23 (s, 2H). TOF MS ES+ (*m*/*z*): (M + H)^+^, calcd for C_35_H_33_BrFN_5_O_5_: 702.1727, found, 702.1703.

*N-(3-Fluoro-4-((6-methoxy-7-(3-(piperidin-1-yl)propoxy)quinolin-4-yl)oxy)phenyl)-1-(4-methoxyphenyl)-4-oxo-1,4-dihydropyridazine-3-carboxamide* (**33**). White solid. 35.2% yield, m.p: 105.4–106.5 °C. ^1^H NMR (400 MHz, DMSO) δ 12.14 (s, 1H), 8.94 (d, *J* = 7.7 Hz, 1H), 8.47 (d, *J* = 4.9 Hz, 1H), 8.02 (d, *J* = 12.7 Hz, 1H), 7.72 (d, *J* = 7.7 Hz, 2H), 7.55 (d, *J* = 8.8 Hz, 2H), 7.49 (t, *J* = 8.8 Hz, 1H), 7.40 (s, 1H), 7.15 (d, *J* = 7.7 Hz, 2H), 6.92 (d, *J* = 7.7 Hz, 1H), 6.49 (d, *J* = 4.9 Hz, 1H), 4.19 (s, 2H), 3.95 (s, 3H), 3.83 (s, 3H), 2.50 (s, 4H), 2.49–2.40 (m, 2H), 2.01 (d, *J* = 6.1 Hz, 2H), 1.54 (s, 4H), 1.41 (s, 2H). ^13^C NMR (101 MHz, DMSO) δ 169.05, 159.97, 159.28, 159.18, 154.73, 152.29, 151.88, 149.57, 148.81, 147.00, 146.35, 141.92, 136.98, 136.36, 124.30, 122.95(2C), 120.66, 116.65, 114.68(2C), 114.49, 108.76, 108.55, 102.10, 99.04, 66.69, 55.77, 55.62, 54.83, 53.76(2C), 25.61, 25.07(2C), 23.65. TOF MS ES+ (*m*/*z*): (M + H)^+^, calcd for C_36_H_36_FN_5_O_6_: 654.2728, found, 654.2705.

*1-(3-Chloro-4-fluorophenyl)-N-(3-fluoro-4-((6-methoxy-7-(3-(piperidin-1-yl)propoxy)quinolin-4-yl)oxy)phenyl)-4-oxo-1,4-dihydropyridazine-3-carboxamide* (**34**). Light yellow solid. 37.9% yield, m.p: 121.7–123.4 °C. ^1^H NMR (400 MHz, DMSO) δ 11.93 (s, 1H), 9.02 (d, *J* = 7.8 Hz, 1H), 8.48 (d, *J* = 5.2 Hz, 1H), 8.17–8.13 (m, 1H), 8.03 (d, *J* = 13.0 Hz, 1H), 7.90–7.84 (m, 1H), 7.71 (t, *J* = 9.0 Hz, 1H), 7.60–7.54 (m, 2H), 7.51 (t, *J* = 8.7 Hz, 1H), 7.40 (s, 1H), 6.94 (d, *J* = 7.9 Hz, 1H), 6.50 (d, *J* = 5.2 Hz, 1H), 4.19 (t, *J* = 6.3 Hz, 2H), 3.96 (s, 3H), 2.43 (t, *J* = 7.0 Hz, 2H), 2.36 (s, 4H), 2.00–1.93 (m, 2H), 1.55–1.47 (m, 4H), 1.39 (s, 2H). TOF MS ES+ (*m*/*z*): (M + H)^+^, calcd for C_35_H_32_ClF_2_N_5_O_5_: 676.2138, found, 676.2109.

*1-(4-Bromo-2-fluorophenyl)-N-(3-fluoro-4-((6-methoxy-7-(3-(piperidin-1-yl)propoxy)quinolin-4-yl)oxy)phenyl)-4-oxo-1,4-dihydropyridazine-3-carboxamide* (**35**). White solid. 42.1% yield, m.p: 105.9–107.4 °C. ^1^H NMR (400 MHz, DMSO) δ 11.79 (s, 1H), 8.74 (d, *J* = 7.9 Hz, 1H), 8.45 (d, *J* = 5.1 Hz, 1H), 7.96 (d, *J* = 11.9 Hz, 2H), 7.74 (t, *J* = 8.3 Hz, 1H), 7.69 (d, *J* = 8.9 Hz, 1H), 7.57–7.50 (m, 2H), 7.48 (t, *J* = 8.8 Hz, 1H), 7.38 (s, 1H), 6.90 (d, *J* = 7.9 Hz, 1H), 6.47 (d, *J* = 5.2 Hz, 1H), 4.17 (t, *J* = 6.3 Hz, 2H), 3.93 (s, 3H), 2.48 (s, 4H), 2.41 (t, *J* = 7.0 Hz, 2H), 1.99–1.89 (m, 2H), 1.55–1.42 (m, 4H), 1.36 (s, 2H). ^13^C NMR (101 MHz, DMSO) δ 168.74, 159.81, 159.16, 154.73, 152.27, 151.96, 149.60, 149.03, 148.80, 146.37, 144.52, 136.87, 130.47, 128.70, 128.53, 124.33, 122.89, 120.60, 120.38, 119.69, 116.64, 114.45, 108.75, 108.51, 102.07, 99.02, 66.81, 55.77, 55.05, 54.06(2C), 26.04, 25.54(2C), 24.08. TOF MS ES+ (*m*/z): (M + H)^+^, calcd for C_35_H_32_BrF_2_N_5_O_5_: 720.1633, found, 720.1612.

*1-(4-Chloro-3-(trifluoromethyl)phenyl)-N-(3-fluoro-4-((6-methoxy-7-(3-(piperidin-1-yl)propoxy)quinolin-4-yl)oxy)phenyl)-4-oxo-1,4-dihydropyridazine-3-carboxamide* (**36**). Light yellow solid. 48.7% yield, m.p: 185.7–186.6 °C. ^1^H NMR (400 MHz, DMSO) δ 11.77 (s, 1H), 9.01 (d, *J* = 8.0 Hz, 1H), 8.37 (d, *J* = 5.1 Hz, 1H), 8.19 (s, 1H), 8.06 (d, *J* = 8.8 Hz, 1H), 7.90 (d, *J* = 8.6 Hz, 2H), 7.46 (d, *J* = 13.6 Hz, 2H), 7.39 (t, *J* = 8.8 Hz, 1H), 7.30 (s, 1H), 6.84 (d, *J* = 7.8 Hz, 1H), 6.39 (d, *J* = 5.0 Hz, 1H), 4.10 (s, 2H), 3.85 (s, 3H), 2.56 (s, 4H), 1.95 (s, 2H), 1.48 (s, 4H), 1.34 (s, 2H), 1.12 (s, 2H). TOF MS ES+ (*m*/*z*): (M + H)^+^, calcd for C_36_H_32_ClF_4_N_5_O_5_: 726.2106, found, 726.2097.

*N-(3-Fluoro-4-((6-methoxy-7-(3-(piperidin-1-yl)propoxy)quinolin-4-yl)oxy)phenyl)-4-oxo-1-phenyl-1,4-dihydropyridazine-3-carboxamide* (**37**). Pure white solid. 38.9% yield, m.p: 152.3–153.1 °C. ^1^H NMR (400 MHz, DMSO) δ 12.02 (s, 1H), 9.03 (d, *J* = 7.7 Hz, 1H), 8.47 (d, *J* = 4.6 Hz, 1H), 8.02 (d, *J* = 12.7 Hz, 1H), 7.81 (d, *J* = 7.6 Hz, 2H), 7.63 (t, *J* = 7.3 Hz, 2H), 7.58–7.46 (m, 4H), 7.39 (s, 1H), 6.94 (d, *J* = 7.7 Hz, 1H), 6.49 (d, *J* = 4.5 Hz, 1H), 4.18 (s, 2H), 3.95 (s, 3H), 2.43 (t, *J* = 6.7 Hz, 2H), 2.35 (s, 4H), 1.96 (d, *J* = 6.0 Hz, 2H), 1.50 (s, 4H), 1.39 (s, 2H). ^13^C NMR (101 MHz, DMSO) δ 168.65, 159.48, 158.66, 154.24, 151.79, 151.46, 149.10, 148.28, 147.12, 145.89, 142.34, 141.26, 136.56, 129.20(2C), 128.11, 123.79, 120.82(2C), 120.02, 116.14, 113.97, 108.27, 108.04, 101.60, 98.54, 66.32, 55.27, 54.53, 53.54(2C), 25.54, 25.02(2C), 23.56. TOF MS ES+ (*m*/*z*): (M + H)^+^, calcd for C_35_H_34_FN_5_O_5_: 624.2622, found, 624.2605.

*N-(3-Fluoro-4-((6-methoxy-7-(3-(4-methylpiperidin-1-yl)propoxy)quinolin-4-yl)oxy)phenyl)-1-(4-fluorophenyl)-4-oxo-1,4-dihydropyridazine-3-carboxamide* (**38**). White solid. 35.8% yield, m.p: 210.1–211.4 °C. ^1^H NMR (400 MHz, DMSO) δ 12.02 (s, 1H), 9.00 (d, *J* = 7.8 Hz, 1H), 8.49 (s, 1H), 8.03 (d, *J* = 12.9 Hz, 1H), 7.88 (d, *J* = 4.8 Hz, 2H), 7.64–7.45 (m, 5H), 7.40 (s, 1H), 6.94 (d, *J* = 7.4 Hz, 1H), 6.50 (s, 1H), 4.19 (s, 2H), 3.96 (s, 3H), 2.87 (d, *J* = 10.5 Hz, 2H), 2.46 (s, 2H), 1.97 (s, 2H), 1.91 (d, *J* = 10.2 Hz, 2H), 1.59 (d, *J* = 11.5 Hz, 2H), 1.33 (s, 1H), 1.15 (d, *J* = 12.2 Hz, 2H), 0.89 (d, *J* = 6.0 Hz, 3H). TOF MS ES+ (*m*/*z*): (M + H)^+^, calcd for C_36_H_35_F_2_N_5_O_5_: 656.2685, found, 656.2673.

*1-(4-Chlorophenyl)-N-(3-fluoro-4-((6-methoxy-7-(3-(4-methylpiperidin-1-yl)propoxy)quinolin-4-yl)oxy)phenyl)-4-oxo-1,4-dihydropyridazine-3-carboxamide* (**39**). Light yellow solid. 51.4% yield, m.p: 220.6–222.1 °C. ^1^H NMR (400 MHz, DMSO) δ 11.96 (s, 1H), 9.02 (d, *J* = 7.8 Hz, 1H), 8.48 (d, *J* = 4.4 Hz, 1H), 8.02 (d, *J* = 12.9 Hz, 1H), 7.86 (d, *J* = 7.5 Hz, 2H), 7.70 (d, *J* = 7.5 Hz, 2H), 7.59–7.46 (m, 3H), 7.39 (s, 1H), 6.94 (d, *J* = 7.9 Hz, 1H), 6.49 (d, *J* = 4.6 Hz, 1H), 4.18 (s, 2H), 3.96 (s, 3H), 2.85 (d, *J* = 10.3 Hz, 2H), 2.44 (t, *J* = 6.4 Hz, 2H), 1.97 (d, *J* = 6.2 Hz, 2H), 1.88 (t, *J* = 11.2 Hz, 2H), 1.57 (d, *J* = 11.7 Hz, 2H), 1.30 (s, 1H), 1.16 (t, *J* = 11.5 Hz, 2H), 0.89 (d, *J* = 6.0 Hz, 3H). ^13^C NMR (101 MHz, DMSO) δ 169.67, 160.51, 159.74, 155.31, 152.87, 152.57, 150.22, 149.38, 148.42, 146.98, 142.18, 137.59, 136.96, 133.59, 130.21(2C), 124.87, 123.60(2C), 120.97, 117.24, 115.06, 109.36, 109.14, 102.71, 99.66, 67.43, 56.37, 55.25, 54.02(2C), 34.57(2C), 30.93, 26.79, 22.34. TOF MS ES+ (*m*/*z*): (M + H)^+^, calcd for C_36_H_35_ClFN_5_O_5_: 672.2389, found, 672.2367.

*1-(4-Bromophenyl)-N-(3-fluoro-4-((6-methoxy-7-(3-(4-methylpiperidin-1-yl)propoxy)quinolin-4-yl)oxy)phenyl)-4-oxo-1,4-dihydropyridazine-3-carboxamide* (**40**). Light yellow solid. 49.8% yield, m.p: 220.7–221.4 °C. ^1^H NMR (400 MHz, DMSO) δ 11.94 (s, 1H), 9.03 (d, *J* = 7.4 Hz, 1H), 8.49 (s, 1H), 8.02 (d, *J* = 12.3 Hz, 1H), 7.84 (d, *J* = 8.7 Hz, 2H), 7.80 (d, *J* = 8.0 Hz, 2H), 7.55 (s, 2H), 7.50 (s, 1H), 7.40 (s, 1H), 6.94 (d, *J* = 7.8 Hz, 1H), 6.51 (s, 1H), 4.20 (s, 2H), 3.96 (s, 3H), 2.92 (s, 2H), 2.53 (s, 2H), 2.00 (s, 4H), 1.61 (d, *J* = 11.7 Hz, 2H), 1.36 (s, 1H), 1.17 (d, *J* = 12.0 Hz, 2H), 0.90 (d, *J* = 5.8 Hz, 3H). TOF MS ES+ (*m*/*z*): (M + H)^+^, calcd for C_36_H_35_BrFN_5_O_5_: 716.1884, found, 716.1863.

*N-(3-Fluoro-4-((6-methoxy-7-(3-(4-methylpiperidin-1-yl)propoxy)quinolin-4-yl)oxy)phenyl)-1-(4-methoxyphenyl)-4-oxo-1,4-dihydropyridazine-3-carboxamide* (**41**). Light yellow solid. 40.3% yield, m.p: 145.1–146.3 °C. ^1^H NMR (400 MHz, DMSO) δ 12.12 (s, 1H), 8.94 (d, *J* = 7.7 Hz, 1H), 8.47 (d, *J* = 5.3 Hz, 1H), 8.02 (d, *J* = 12.9 Hz, 1H), 7.73 (d, *J* = 8.7 Hz, 2H), 7.54 (s, 2H), 7.49 (t, *J* = 8.3 Hz, 1H), 7.39 (s, 1H), 7.16 (d, *J* = 7.4 Hz, 2H), 6.92 (d, *J* = 8.0 Hz, 1H), 6.50 (s, 1H), 4.18 (s, 2H), 3.95 (s, 3H), 3.84 (s, 3H), 2.85 (d, *J* = 9.0 Hz, 2H), 2.44 (d, *J* = 6.5 Hz, 2H), 1.97 (d, *J* = 6.9 Hz, 2H), 1.87 (t, *J* = 11.4 Hz, 2H), 1.57 (d, *J* = 11.9 Hz, 2H), 1.30 (s, 1H), 1.19–1.11 (m, 2H), 0.88 (d, *J* = 6.3 Hz, 3H). TOF MS ES+ (*m*/*z*): (M + H)^+^, calcd for C_37_H_38_FN_5_O_6_: 668.2884, found, 668.2836.

*1-(3-Chloro-4-fluorophenyl)-N-(3-fluoro-4-((6-methoxy-7-(3-(4-methylpiperidin-1-yl)propoxy)quinolin-4-yl)oxy)phenyl)-4-oxo-1,4-dihydropyridazine-3-carboxamide* (**42**). Light yellow solid. 41.5% yield, m.p: 112.5–113.7 °C. ^1^H NMR (400 MHz, DMSO) δ 11.94 (s, 1H), 9.03 (d, *J* = 7.8 Hz, 1H), 8.49 (d, *J* = 5.1 Hz, 1H), 8.15 (d, *J* = 4.0 Hz, 1H), 8.03 (d, *J* = 12.7 Hz, 1H), 7.88 (d, *J* = 9.0 Hz, 1H), 7.71 (t, *J* = 8.8 Hz, 1H), 7.57 (d, *J* = 13.1 Hz, 2H), 7.51 (t, *J* = 8.8 Hz, 1H), 7.42 (s, 1H), 6.94 (d, *J* = 7.8 Hz, 1H), 6.50 (d, *J* = 5.0 Hz, 1H), 4.22 (s, 2H), 3.97 (s, 3H), 3.10 (s, 2H), 2.75 (s, 2H), 2.09 (s, 2H), 1.67 (d, *J* = 12.3 Hz, 2H), 1.30 (s, 1H), 1.25 (d, *J* = 12.3 Hz, 4H), 0.91 (d, *J* = 6.3 Hz, 3H). TOF MS ES+ (*m*/*z*): (M + H)^+^, calcd for C_36_H_34_ClF_2_N_5_O_5_: 690.2295, found, 690.2273.

*1-(4-Bromo-2-fluorophenyl)-N-(3-fluoro-4-((6-methoxy-7-(3-(4-methylpiperidin-1-yl)propoxy)quinolin-4-yl)oxy)phenyl)-4-oxo-1,4-dihydropyridazine-3-carboxamide* (**43**). White solid. 35.3% yield, m.p: 115.8–117.3 °C. ^1^H NMR (400 MHz, DMSO) δ 11.78 (s, 1H), 8.74 (d, *J* = 7.9 Hz, 1H), 8.46 (s, 1H), 7.96 (d, *J* = 12.2 Hz, 2H), 7.79–7.66 (m, 2H), 7.52 (s, 2H), 7.51–7.44 (m, 1H), 7.37 (s, 1H), 6.90 (d, *J* = 7.8 Hz, 1H), 6.47 (d, *J* = 5.1 Hz, 1H), 4.17 (t, *J* = 5.9 Hz, 2H), 3.93 (s, 3H), 2.85 (d, *J* = 10.6 Hz, 2H), 2.45 (s, 2H), 1.99–1.93 (m, 2H), 1.90 (d, *J* = 11.9 Hz, 2H), 1.56 (d, *J* = 12.3 Hz, 2H), 1.22 (d, *J* = 10.2 Hz, 1H), 1.13 (d, *J* = 12.3 Hz, 2H), 0.87 (d, *J* = 6.3 Hz, 3H). ^13^C NMR (101 MHz, DMSO) δ 168.76, 159.78, 159.16, 154.73, 153.28, 151.96, 149.60, 148.97, 148.78, 146.38, 136.87, 130.37, 128.71, 128.70, 128.52, 124.32, 122.97, 122.89, 120.61, 120.38, 119.71, 116.64, 108.76, 108.52, 102.08, 99.03, 66.80, 55.76, 54.67, 53.43(2C), 33.93(2C), 30.33, 26.16, 21.77. TOF MS ES+ (*m*/*z*): (M + H)^+^, calcd for C_36_H_34_BrF_2_N_5_O_5_: 734.1790, found, 734.1773.

*1-(4-Chloro-3-(trifluoromethyl)phenyl)-N-(3-fluoro-4-((6-methoxy-7-(3-(4-methylpiperidin-1-yl)propoxy)quinolin-4-yl)oxy)phenyl)-4-oxo-1,4-dihydropyridazine-3-carboxamide* (**44**). Light yellow solid. 38.7% yield, m.p: 137.7–138.6 °C. ^1^H NMR (400 MHz, DMSO) δ 11.77 (s, 1H), 9.02 (d, *J* = 7.9 Hz, 1H), 8.37 (d, *J* = 5.3 Hz, 1H), 8.19 (s, 1H), 8.06 (d, *J* = 8.4 Hz, 1H), 7.90 (d, *J* = 8.5 Hz, 2H), 7.50–7.42 (m, 2H), 7.39 (t, *J* = 8.9 Hz, 1H), 7.30 (s, 1H), 6.84 (d, *J* = 8.2 Hz, 1H), 6.39 (d, *J* = 5.3 Hz, 1H), 4.09 (s, 2H), 3.85 (s, 3H), 2.91 (s, 2H), 1.99 (d, *J* = 48.7 Hz, 4H), 1.53 (d, *J* = 10.0 Hz, 2H), 1.30 (s, 1H), 1.12 (s, 4H), 0.79 (d, *J* = 6.3 Hz, 3H). ^13^C NMR (101 MHz, DMSO) δ 169.66, 160.26, 159.66, 155.22, 152.77, 152.34, 150.03, 149.30, 148.44, 146.84, 142.26, 142.08, 137.47, 136.98, 133.53, 130.85, 127.13, 124.79, 121.39, 121.33, 120.70, 117.17, 115.00, 109.06, 102.61, 101.99, 99.53, 67.12, 56.25, 54.85, 53.52(2C), 33.67(2C), 30.28, 26.04, 22.04. TOF MS ES+ (*m*/*z*): (M + H)^+^, calcd for C_37_H_34_ClF_4_N_5_O_5_: 740.2263, found, 740.2237.

*N-(3-Fluoro-4-((6-methoxy-7-(3-(4-methylpiperidin-1-yl)propoxy)quinolin-4-yl)oxy)phenyl)-4-oxo-1-phenyl-1,4-dihydropyridazine-3-carboxamide* (**45**). Pure white solid. 44.1% yield, m.p: 148.5–150.1 °C. ^1^H NMR (400 MHz, DMSO) δ 12.02 (s, 1H), 9.03 (d, *J* = 7.7 Hz, 1H), 8.47 (d, *J* = 5.1 Hz, 1H), 8.02 (d, *J* = 13.1 Hz, 1H), 7.82 (d, *J* = 7.8 Hz, 2H), 7.63 (t, *J* = 7.7 Hz, 2H), 7.51 (dd, *J* = 17.2, 8.4 Hz, 4H), 7.39 (s, 1H), 6.94 (d, *J* = 7.8 Hz, 1H), 6.49 (d, *J* = 4.8 Hz, 1H), 4.18 (s, 2H), 3.95 (s, 3H), 2.86 (d, *J* = 10.9 Hz, 2H), 2.45 (t, *J* = 6.7 Hz, 2H), 2.01–1.94 (m, 2H), 1.89 (t, *J* = 11.7 Hz, 2H), 1.57 (d, *J* = 11.2 Hz, 2H), 1.32 (s, 1H), 1.20–1.11 (m, 2H), 0.88 (d, *J* = 6.2 Hz, 3H). ^13^C NMR (101 MHz, DMSO) δ 169.63, 160.54, 159.68, 155.25, 152.44, 150.13, 149.33, 148.26, 146.90, 143.37, 142.29, 137.48, 136.85, 130.23(2C), 129.13, 124.81, 121.86(2C), 121.00, 117.17, 115.02, 109.29, 109.12, 102.67, 99.60, 67.27, 56.31, 55.03, 53.76(2C), 34.12(2C), 30.58, 26.42, 22.16. TOF MS ES+ (*m*/*z*): (M + H)^+^, calcd for C_36_H_36_FN_5_O_5_: 638.2779, found, 638.2754.

*N-(3-Fluoro-4-((6-methoxy-7-(3-(4-methylpiperazin-1-yl)propoxy)quinolin-4-yl)oxy)phenyl)-1-(4-fluorophenyl)-4-oxo-1,4-dihydropyridazine-3-carboxamide* (**46**). White solid. 36.1% yield, m.p: 184.6–185.7 °C. ^1^H NMR (400 MHz, DMSO) δ 12.03 (s, 1H), 9.00 (d, *J* = 7.7 Hz, 1H), 8.48 (d, *J* = 4.7 Hz, 1H), 8.02 (d, *J* = 12.5 Hz, 1H), 7.88 (s, 2H), 7.55 (s, 2H), 7.54–7.44 (m, 3H), 7.40 (s, 1H), 6.94 (d, *J* = 7.7 Hz, 1H), 6.50 (s, 1H), 4.19 (s, 2H), 3.96 (s, 3H), 2.47 (s, 2H), 2.42 (d, *J* = 42.1 Hz, 8H), 2.17 (s, 3H), 1.97 (s, 2H). ^13^C NMR (101 MHz, DMSO) δ 168.57, 162.32, 159.87, 159.44, 158.67, 154.24, 151.44, 149.09, 148.31, 147.05, 145.88, 141.53, 138.91, 136.44, 123.82, 123.38(2C), 123.30, 119.95, 116.12(2C), 115.88, 113.96, 108.03, 101.61, 98.53, 66.20, 55.27, 54.02(2C), 53.73, 51.91(2C), 44.90, 25.46. TOF MS ES+ (*m*/*z*): (M + H)^+^, calcd for C_35_H_34_F_2_N_6_O_5_: 657.2637, found, 657.2612.

*1-(4-Chlorophenyl)-N-(3-fluoro-4-((6-methoxy-7-(3-(4-methylpiperazin-1-yl)propoxy)quinolin-4-yl)oxy)phenyl)-4-oxo-1,4-dihydropyridazine-3-carboxamide* (**47**). White solid. 59.1% yield, m.p: 163.5–165.3 °C. ^1^H NMR (400 MHz, DMSO) δ 11.95 (s, 1H), 9.03 (d, *J* = 7.1 Hz, 1H), 8.48 (d, *J* = 4.5 Hz, 1H), 8.02 (d, *J* = 13.1 Hz, 1H), 7.86 (d, *J* = 7.2 Hz, 2H), 7.71 (d, *J* = 8.3 Hz, 2H), 7.59–7.47 (m, 3H), 7.40 (s, 1H), 6.94 (d, *J* = 7.8 Hz, 1H), 6.50 (s, 1H), 4.19 (s, 2H), 3.96 (s, 3H), 2.46 (d, *J* = 6.6 Hz, 2H), 2.35–2.22 (s, 8H), 2.15 (s, 3H), 1.97 (s, 2H). TOF MS ES+ (*m*/*z*): (M + H)^+^, calcd for C_35_H_34_ClFN_6_O_5_: 673.2341, found, 673.2315.

*1-(4-Bromophenyl)-N-(3-fluoro-4-((6-methoxy-7-(3-(4-methylpiperazin-1-yl)propoxy)quinolin-4-yl)oxy)phenyl)-4-oxo-1,4-dihydropyridazine-3-carboxamide* (**48**). White solid. 38.2% yield, m.p: 159.7–161.2 °C. ^1^H NMR (400 MHz, DMSO) δ 11.95 (s, 1H), 9.03 (d, *J* = 7.9 Hz, 1H), 8.49 (d, *J* = 5.2 Hz, 1H), 8.03 (d, *J* = 12.6 Hz, 1H), 7.82 (m, *J* = 8.9 Hz, 4H), 7.57 (d, *J* = 10.4 Hz, 2H), 7.54–7.48 (m, 1H), 7.42 (s, 1H), 6.94 (d, *J* = 7.8 Hz, 1H), 6.51 (d, *J* = 5.1 Hz, 1H), 4.21 (s, 2H), 3.97 (s, 3H), 2.78 (s, 4H), 2.60 (s, 4H), 2.47 (s, 2H), 2.03 (d, *J* = 6.1 Hz, 2H), 1.23 (s, 3H). TOF MS ES+ (*m*/*z*): (M + H)^+^, calcd for C_35_H_34_BrFN_6_O_5_: 717.1836, found, 717.1804.

*1-(4-((l1-Oxidanyl)-l5-methyl)phenyl)-N-(3-fluoro-4-((6-methoxy-7-(3-(4-methylpiperazin-1-yl)propoxy)quinolin-4-yl)oxy)phenyl)-4-oxo-1,4-dihydropyridazine-3-carboxamide* (**49**). Light yellow solid. 47.5% yield, m.p: 167.5–169.1 °C. ^1^H NMR (400 MHz, DMSO) δ 12.02 (s, 1H), 8.83 (d, *J* = 7.7 Hz, 1H), 8.36 (d, *J* = 4.0 Hz, 1H), 7.91 (d, *J* = 12.9 Hz, 1H), 7.62 (d, *J* = 8.8 Hz, 2H), 7.44 (d, *J* = 10.3 Hz, 2H), 7.39 (t, *J* = 8.7 Hz, 1H), 7.28 (s, 1H), 7.05 (d, *J* = 8.7 Hz, 2H), 6.82 (d, *J* = 7.9 Hz, 1H), 6.39 (s, 1H), 4.08 (s, 2H), 3.84 (s, 3H), 3.73 (s, 3H), 2.35 (d, *J* = 6.8 Hz, 2H), 2.37–2.14 (s, 8H), 2.06 (s, 3H), 1.86 (s, 2H). ^13^C NMR (101 MHz, DMSO) δ 168.79, 159.80, 159.17, 155.77, 154.70, 153.23, 152.26, 151.91, 149.56, 148.93, 148.78, 146.33, 144.46, 137.06, 128.67, 128.49, 124.27, 122.96, 120.57, 120.34, 119.70, 116.66, 114.45, 108.46, 102.05, 99.00, 66.65, 66.18(3C), 55.74, 54.79, 53.34(3C), 25.65. TOF MS ES+ (*m*/*z*): (M + H)^+^, calcd for C_36_H_37_FN_6_O_6_: 669.2837, found, 669.2801.

*1-(3-Chloro-4-fluorophenyl)-N-(3-fluoro-4-((6-methoxy-7-(3-(4-methylpiperazin-1-yl)propoxy)quinolin-4-yl)oxy)phenyl)-4-oxo-1,4-dihydropyridazine-3-carboxamide* (**50**). Light yellow solid. 35.4% yield, m.p: 144.7–146.2 °C. ^1^H NMR (400 MHz, DMSO) δ 11.92 (s, 1H), 9.02 (d, *J* = 7.9 Hz, 1H), 8.48 (d, *J* = 5.1 Hz, 1H), 8.14 (dd, *J* = 6.3, 2.7 Hz, 1H), 8.02 (d, *J* = 12.6 Hz, 1H), 7.94–7.83 (m, 1H), 7.71 (t, *J* = 9.0 Hz, 1H), 7.61–7.54 (m, 2H), 7.51 (t, *J* = 8.8 Hz, 1H), 7.40 (s, 1H), 6.94 (d, *J* = 7.9 Hz, 1H), 6.50 (d, *J* = 5.2 Hz, 1H), 4.19 (t, *J* = 6.2 Hz, 2H), 3.96 (s, 3H), 2.51 (s, 2H), 2.49–2.45 (m, 2H), 2.35 (s, 4H), 2.16 (s, 3H), 2.02–1.93 (m, 2H), 1.37–1.17 (m, 2H). ^13^C NMR (101 MHz, DMSO) δ 169.66, 160.22, 159.66, 158.57, 156.09, 155.23, 152.47, 150.12, 149.27, 148.00, 146.88, 142.31, 140.17, 137.44, 137.03, 124.75, 124.24, 122.75, 120.84, 118.40, 118.18, 117.19, 115.00, 109.04, 102.62, 99.57, 67.26, 56.26, 55.24(2C), 54.82, 53.19(2C), 46.18, 26.54. TOF MS ES+ (*m*/*z*): (M + H)^+^, calcd for C_35_H_33_ClF_2_N_6_O_5_: 691.2247, found, 691.2255.

*1-(4-Bromo-2-fluorophenyl)-N-(3-fluoro-4-((6-methoxy-7-(3-(4-methylpiperazin-1-yl)propoxy)quinolin-4-yl)oxy)phenyl)-4-oxo-1,4-dihydropyridazine-3-carboxamide* (**51**). White solid. 39.8% yield, m.p: 103.1–104.7 °C. ^1^H NMR (400 MHz, DMSO) δ 11.82 (s, 1H), 8.76 (dd, *J* = 7.8, 1.8 Hz, 1H), 8.48 (d, *J* = 5.2 Hz, 1H), 8.00 (s, 1H), 7.97 (d, *J* = 1.9 Hz, 1H), 7.77 (t, *J* = 8.4 Hz, 1H), 7.71 (d, *J* = 8.5 Hz, 1H), 7.55 (d, *J* = 8.8 Hz, 2H), 7.49 (t, *J* = 8.8 Hz, 1H), 7.40 (s, 1H), 6.91 (d, *J* = 7.9 Hz, 1H), 6.49 (d, *J* = 5.1 Hz, 1H), 4.20 (t, *J* = 6.4 Hz, 2H), 3.96 (s, 3H), 2.67 (s, 4H), 2.57 (s, 4H), 2.39 (s, 3H), 2.05–1.95 (m, 2H), 1.23 (s, 2H). ^13^C NMR (101 MHz, DMSO) δ 169.21, 160.34, 159.67, 156.32, 155.21, 153.78, 152.76, 152.43, 150.10, 149.60, 149.32, 146.85, 144.99, 129.19, 129.05, 124.79, 123.46, 121.10, 120.87, 120.16, 117.16, 114.99, 109.04, 102.62, 99.56, 67.16, 56.29, 54.55(3C), 52.32(2C), 45.29, 26.33. TOF MS ES+ (*m*/*z*): (M + H)^+^, calcd for C_35_H_33_BrF_2_N_6_O_5_: 735.1742, found, 735.1713.

*1-(4-Chloro-3-(trifluoromethyl)phenyl)-N-(3-fluoro-4-((6-methoxy-7-(3-(4-methylpiperazin-1-yl)propoxy)quinolin-4-yl)oxy)phenyl)-4-oxo-1,4-dihydropyridazine-3-carboxamide* (**52**). Light yellow solid. 43.7% yield, m.p: 211.7–212.6 °C. ^1^H NMR (400 MHz, DMSO) δ 11.77 (s, 1H), 9.03 (t, *J* = 8.1 Hz, 1H), 8.37 (d, *J* = 5.2 Hz, 1H), 8.19 (s, 1H), 8.06 (d, *J* = 8.7 Hz, 1H), 7.93–7.88 (m, 2H), 7.48–7.42 (m, 2H), 7.42–7.36 (m, 1H), 7.30 (d, *J* = 7.9 Hz, 1H), 6.85 (t, *J* = 8.0 Hz, 1H), 6.38 (d, *J* = 5.2 Hz, 1H), 4.09 (d, *J* = 6.5 Hz, 2H), 3.85 (d, *J* = 7.9 Hz, 3H), 2.42 (d, *J* = 6.3 Hz, 4H), 2.40 (s, 4H), 2.20 (d, *J* = 7.6 Hz, 3H), 1.89 (d, *J* = 6.9 Hz, 2H), 1.12 (s, 2H). ^13^C NMR (101 MHz, DMSO) δ 169.16, 159.80, 159.17, 154.73, 152.28, 151.94, 149.61, 148.80, 148.03, 146.38, 141.60, 139.90, 136.99, 136.53, 133.04, 132.81, 130.34, 126.64, 125.45, 124.27, 120.86, 120.19, 116.70, 114.51, 108.58, 102.14, 99.08, 66.67, 55.78, 54.02(3C), 51.78(2C), 44.76, 25.85. TOF MS ES+ (*m*/*z*): (M + H)^+^, calcd for C_36_H_33_ClF_4_N_6_O_5_: 741.2215, found, 741.2216.

*N-(3-Fluoro-4-((6-methoxy-7-(3-(4-methylpiperazin-1-yl)propoxy)quinolin-4-yl)oxy)phenyl)-4-oxo-1-phenyl-1,4-dihydropyridazine-3-carboxamide* (**53**). Pure white solid. 45.2% yield, m.p: 151.3–152.1°C. ^1^H NMR (400 MHz, DMSO) δ 12.02 (s, 1H), 9.03 (d, *J* = 7.8 Hz, 1H), 8.47 (d, *J* = 4.8 Hz, 1H), 8.02 (d, *J* = 12.0 Hz, 1H), 7.82 (d, *J* = 7.4 Hz, 2H), 7.63 (t, *J* = 7.4 Hz, 2H), 7.51 (dd, *J* = 16.5, 8.1 Hz, 4H), 7.39 (s, 1H), 6.94 (d, *J* = 7.7 Hz, 1H), 6.49 (d, *J* = 4.7 Hz, 1H), 4.18 (s, 2H), 3.95 (s, 3H), 2.45 (d, *J* = 6.7 Hz, 2H), 2.42–2.22 (s, 8H), 2.15 (s, 3H), 1.97 (d, *J* = 5.9 Hz, 2H). ^13^C NMR (101 MHz, DMSO) δ 169.14, 160.02, 159.18, 154.75, 152.30, 151.97, 149.63, 148.81, 147.69, 146.40, 142.87, 141.78, 137.07, 129.72(2C), 128.62, 124.30, 121.35(2C), 120.52, 116.67, 114.50, 108.79, 108.57, 102.14, 99.08, 66.74, 55.80, 54.62(2C), 54.27, 52.53(2C), 45.52, 26.02. TOF MS ES+ (*m*/*z*): (M + H)^+^, calcd for C_35_H_35_FN_6_O_5_: 639.2731, found, 639.2689.

### 3.3. Cytotoxicity Assay In Vitro

The cytotoxic activities of target compounds (**22**–**53**) were evaluated with A549, HepG2, and MCF-7 cell lines by the standard MTT assay in vitro, with compounds c-MET inhibitors Foretinib as positive control. The cancer cell lines were cultured in minimum essential medium (MEM) supplement with 10% fetal bovine serum (FBS). Approximately 4 × 103 cells, suspended in MEM medium, were plated onto each well of a 96-well plate and incubated in 5% CO_2_ at 37 °C for 24 h. The test compounds at indicated final concentrations were added to the culture medium and the cell cultures were continued for 72 h. Fresh MTT was added to each well at a terminal concentration of 5 μg/mL and incubated with cells at 37 °C for 4 h. The formazan crystals were dissolved in 100 μL DMSO each well, and the absorbency at 492 nm (for absorbance of MTT formazan) and 630 nm (for the reference wavelength) was measured with the ELISA reader. All of the compounds were tested two times in each of the cell lines. The results expressed as inhibition rates or IC_50_ (half-maximal inhibitory concentration) were the averages of two determinations and calculated by using the Bacus Laboratories Incorporated Slide Scanner (Bliss) software (the Bacus Laboratories Inc. Slide Scanner (BLISS) system, Lombard, IL, USA). [20].

### 3.4. Tyrosine Kinases Assay In Vitro

The selected compounds (**22**–**53**) are tested for their activity against c-Met Tyrosine kinases through the mobility shift assay [8,9]. All kinase assays were performed in 96-well plates in a 50 µL reaction volume. The kinase buffer contains 50 mM HEPES, pH 7.5, 10 mM MgCl_2_, 0.0015% Brij-35 and 2 mM DTT. The stop buffer contains 100 mM HEPES, pH 7.5, 0.015% Brij-35, 0.2% Coating Reagent 3 and 50 mM EDTA. Dilute the compounds to 500 µM by 100% DMSO, then transfer 10 µL of compound to a new 96-well plate as the intermediate plate, add 90 µL kinase buffer to each well. Transfer 5 µL of each well of the intermediate plate to 384-well plates. The following amounts of enzyme and substrate were used per well: kinase base buffer, FAM-labeled peptide, ATP and enzyme solution. Wells containing the substrate, enzyme, DMSO without compound were used as DMSO control. Wells containing just the substrate without enzyme were used as low control. Incubate at room temperature for 10 min. Add 10 µL peptide solution to each well. Incubate at 28 °C for specified period of time and stop reaction by 25 µL stop buffer. At last collect data on Caliper program and convert conversion values to inhibition values. Percent inhibition = (max − conversion)/(max − min) × 100. ‘max’ stands for DMSO control; ‘min’ stands for low control [20]. 

### 3.5. Observation of Nuclear Morphology

The cancer cell lines were cultured in minimum essential medium (MEM) supplement with 10% fetal bovine serum (FBS). Approximately 2 × 104 cells, suspended in MEM medium, were plated onto each well of a 24-well plate and incubated in 5% CO_2_ at 37 °C for 24 h. Then the medium was removed, and 1 mL drug-free medium and 1mL medium with the test compound **7c** at indicated final concentrations were added to each well in control group and test group respectively and the cell cultures were continued for 12 h. While the medium was removed, AO-PBS buffer was added to each well at a terminal concentration of 10 µg/mL and stained in dark for 10 min. Each well was washed with PBS buffer three times and observed under a fluorescence microscope.

### 3.6. Docking Studies

For docking purposes, the three-dimensional structure of the c-Met (PDB code: 3LQ8) was obtained from RCSB Protein Data Bank [10]. Hydrogen atoms were added to the structure allowing for appropriate ionization at physiological pH and the water was removed. The protonated state of several important residues were adjusted by using AutoDock vina v1.02 in favor of forming reasonable hydrogen bond with the ligand. And using AutoDock vina v1.02 to produce nine ligand conformation, the best molecular conformation was used as a ligand that complex bonding affinity score was −32.83 kcal/mol. Molecular docking of **53** into the 3D c-Met complex structure (PDB code: 3LQ8) was carried out using the Discovery Studio 3.5, as implemented through the graphical user interface LibDock protocol. Using the receptor-ligand interaction part to defined the 3LQ8 as receptor to show the interaction between the **53** and c-Met, then display the residues that contact with **53** and hide others to produce the Figure 4a after adding the activity pocket. Follow by point the “show 2D diagram” to obtain the Figure 4b. All calculations were performed on Silicon Graphics workstation [20].

## 4. Conclusions

In summary, we designed and synthesized a series of 6,7-disubstituted-4-phenoxyquinoline derivatives bearing pyridazinone skeleton as potential c-Met kinase inhibitors and evaluated for the IC_50_ values against three cancer cell lines and enzymatic. Eight of them are equal or more active than positive control Foretinib against one or more cell lines and enzymatic. The most promising compound **53** showed superior activity to Foretinib, which possessed excellent c-Met kinase inhibition on a single digital nanomolar level (IC_50_ = 0.6 nM), and cancer cells of A549 (IC_50_ = 0.003 µM), HepG2 (IC_50_ = 0.49 µM) and MCF-7 (IC_50_ = 0.006 µM). Moreover, SARs and docking studies indicated that 6,7-disubstituted-4-phenoxyquinoline derivatives bearing pyridazinone were favorable to the activity. What’s more, the morpholino group was substituted with other water-soluble substituents to favor this activity. In particular, the 4-methylpiperazinyl group was most active. According to the result of AO single staining, it’s claimed that the **53** could induce remarkable apoptosis of HepG2 cells. And further study will be carried out to identify the exact action mechanism in near future.

## Figures and Tables

**Table 1:**
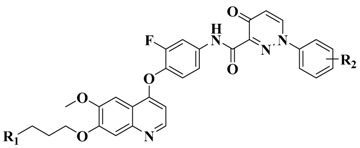
c-Met Enzymatic and Cellular Activities of the target compounds **22**–**53**.

Compounds	R_1_	R_2_	IC_50_^a^ (nM)	IC_50_ (µM)		
			c-Met	A549	HepG2	MCF-7
22	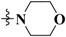	4-F	47.0	0.25 ± 0.01	2.23 ± 0.35	0.21 ± 0.02
23		4-Cl	>500	NA ^b^	NA	NA
24		4-Br	>500	NA	NA	NA
25		4-OCH_3_	233.0	4.24 ± 0.62	6.75 ± 0.82	27.24 ± 1.44
26		3-Cl-4F	352.0	3.70 ± 0.57	NA	5.96 ± 0.77
27		2-F-4Br	478.0	NA	NA	NA
28		3-CF_3_-4Cl	382.0	5.25 ± 0.72	NA	7.49 ± 0.87
29		H	16.0	0.20 ± 0.02	2.27 ± 0.36	0.03 ± 0.01
30	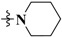	4-F	17.0	0.31 ± 0.01	0.99 ± 0.01	0.69 ± 0.02
31		4-Cl	78.0	0.57 ± 0.02	0.69 ± 0.03	1.16 ± 0.06
32		4-Br	246.0	2.89 ± 0.46	1.75 ± 0.24	2.45 ± 0.39
33		4-OCH_3_	56.0	2.78 ± 0.44	0.63 ± 0.08	2.35 ± 0.37
34		3-Cl-4F	2.0	0.36 ± 0.04	1.28 ± 0.11	0.15 ± 0.04
35		2-F-4Br	8.0	1.36 ± 0.19	3.10 ± 0.49	1.19 ± 0.08
36		3-CF_3_-4Cl	11.0	0.79 ± 0.06	1.80 ± 0.26	0.73 ± 0.08
37		H	4.0	0.06 ± 0.01	0.18 ± 0.01	0.03 ± 0.01
38	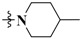	4-F	>500	28.89 ± 1.46	47.23 ± 1.67	22.47 ± 1.35
39		4-Cl	169.0	12.43 ± 1.09	7.81 ± 0.89	14.14 ± 1.15
40		4-Br	345.0	13.47 ± 1.13	8.84 ± 0.95	9.74 ± 0.99
41		4-OCH_3_	0.9	0.02 ± 0.01	0.008 ± 0.001	0.02 ± 0.01
42		3-Cl-4F	1.2	0.005 ± 0.001	1.03 ± 0.01	0.007 ± 0.001
43		2-F-4Br	2.0	0.03 ± 0.01	0.003 ± 0.001	0.005 ± 0.001
44		3-CF_3_-4Cl	19.0	0.15 ± 0.03	0.65 ± 0.05	5.96 ± 0.07
45		H	187.0	2.95 ± 0.47	4.66 ± 0.67	2.01 ± 0.30
46	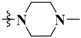	4-F	39.0	1.64 ± 0.22	7.51 ± 0.87	1.66 ± 0.22
47		4-Cl	145.0	19.99 ± 1.30	14.25 ± 1.15	21.08 ± 1.32
48		4-Br	>500	NA	NA	NA
49		4-OCH_3_	78.0	9.12 ± 0.96	3.68 ± 0.57	8.12 ± 0.91
50		3-Cl-4F	2.0	0.02 ± 0.01	2.46 ± 0.39	0.02 ± 0.01
51		2-F-4Br	123.0	5.63 ± 0.75	6.39 ± 0.81	5.43 ± 0.74
52		3-CF_3_-4Cl	0.8	0.04 ± 0.01	0.33 ± 0.03	0.15 ± 0.02
53		H	0.6	0.003 ± 0.001	0.49 ± 0.03	0.006 ± 0.001
Foretinib ^c^	-	-	1.8	0.26 ± 0.03	0.84 ± 0.07	2.76 ± 0.53

^a^ The values are an average of two separate determinations. ^b^ NA: Not active (IC_50_ > 50 µM). ^c^ Used as a positive control.

## Supplementary Materials

Supplementary File 1
