# Supplementary material for: Design, Synthesis and Biological Evaluation of 6,7-Disubstituted-4-phenoxyquinoline Derivatives Bearing Pyridazinone Moiety as c-Met Inhibitors
Source: Molecules. 2018 Jun 26;23(7):1543. doi: 10.3390/molecules23071543 (PMC6099740; doi:10.3390/molecules23071543)
Supplement: Supplementary File 1 [file molecules-23-01543-s001.pdf]

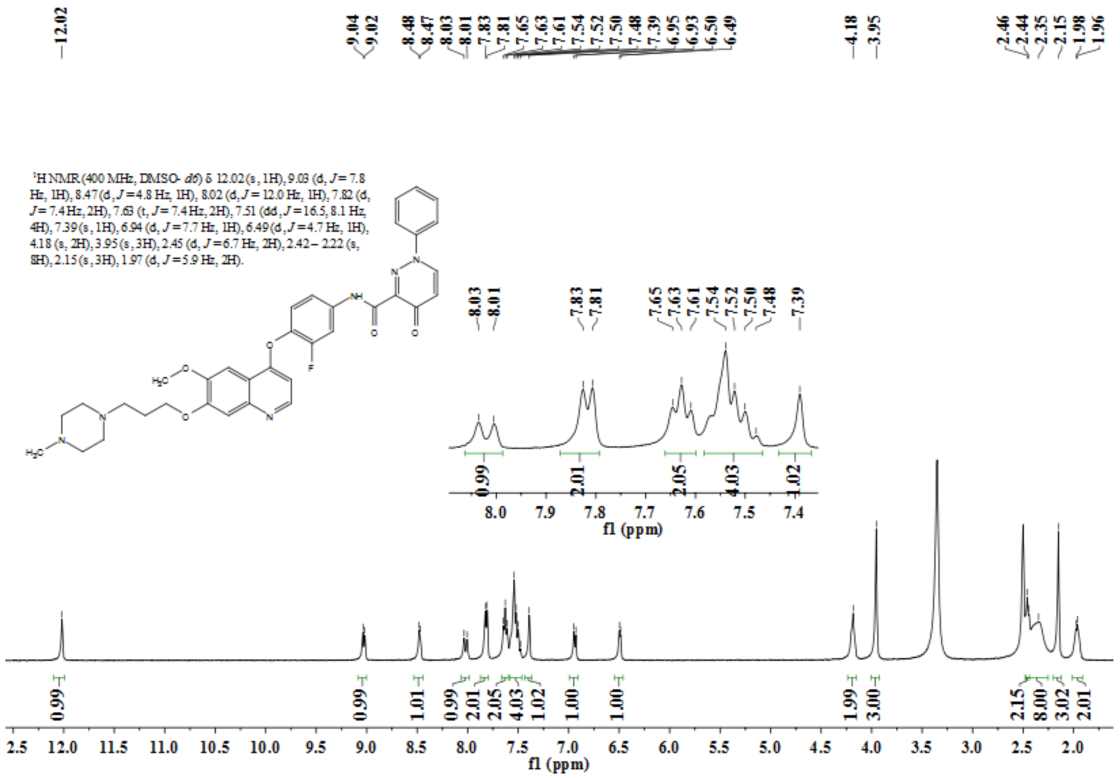

Fig.1 ^{1}H NMR spectrum of compound **53**

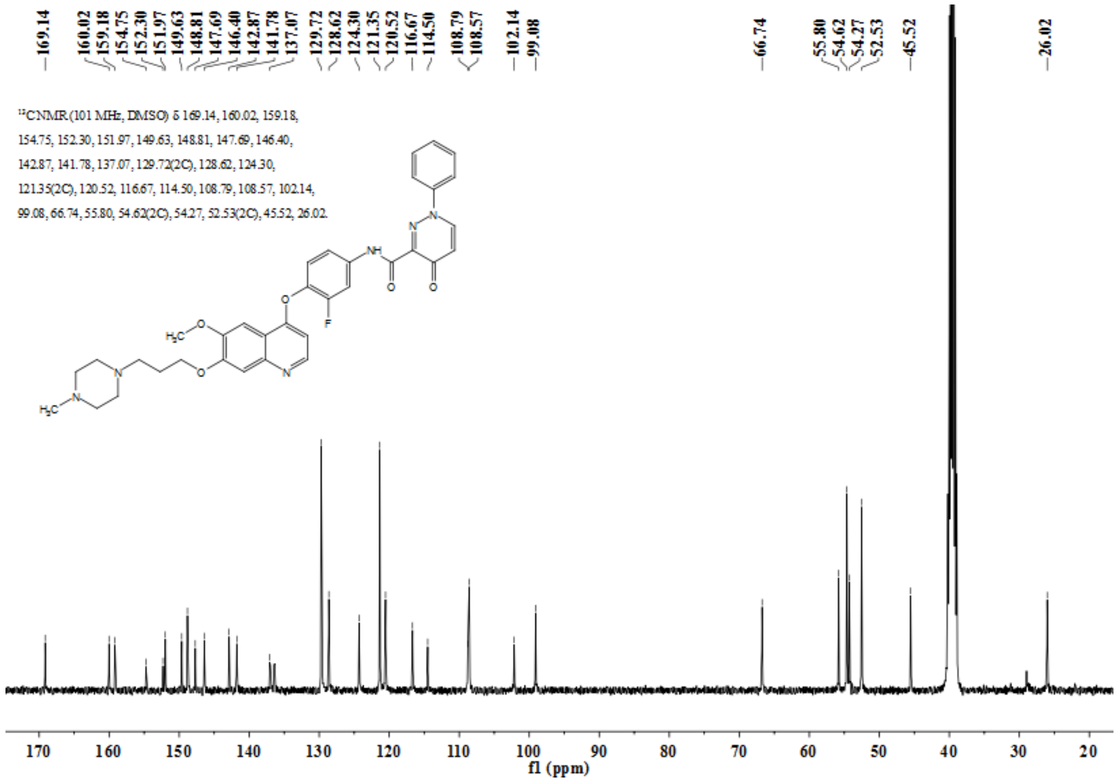

Fig.2 ^{13}C NMR spectrum of compound **53**

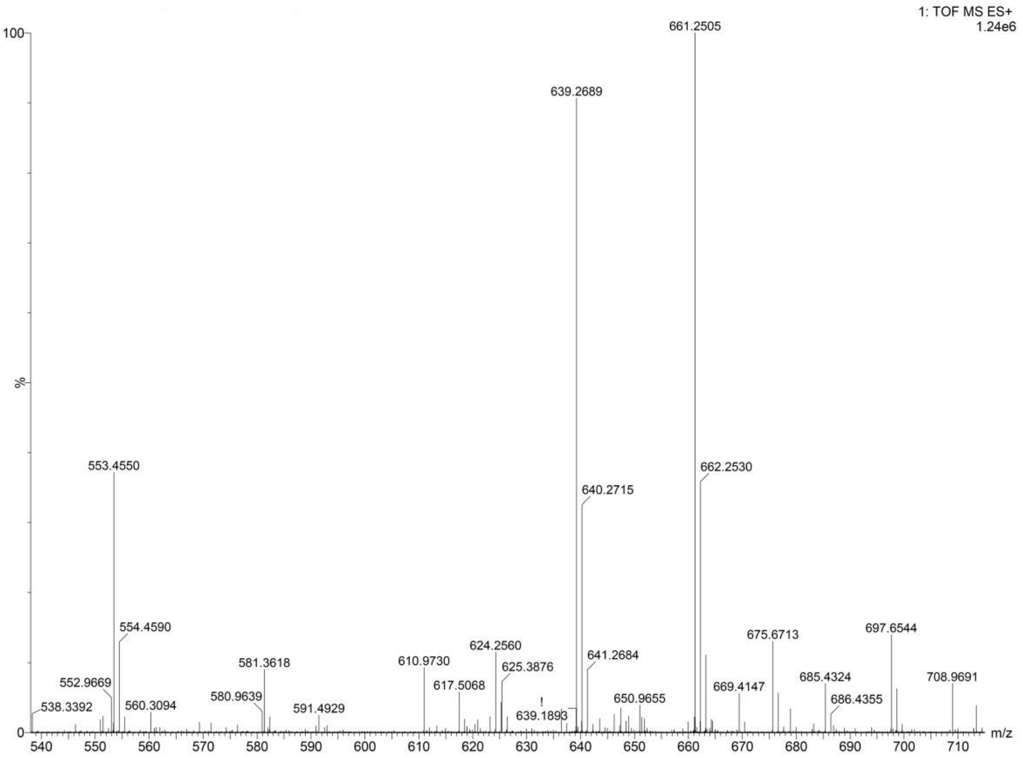

Fig.3 LC-MS spectrum of compound **53**
